# Excessive energy expenditure due to acute physical restraint disrupts *Drosophila* motivational feeding response

**DOI:** 10.1038/s41598-021-03575-3

**Published:** 2021-12-17

**Authors:** Jacob Gordon, Pavel Masek

**Affiliations:** Department of Biological Sciences, Binghamton University, 4400 Vestal Parkway East, Binghamton, NY 13902 USA

**Keywords:** Neuroscience, Feeding behaviour, Motivation, Stress and resilience, Metabolism

## Abstract

To study the behavior of *Drosophila*, it is often necessary to restrain and mount individual flies. This requires removal from food, additional handling, anesthesia, and physical restraint. We find a strong positive correlation between the length of time flies are mounted and their subsequent reflexive feeding response, where one hour of mounting is the approximate motivational equivalent to ten hours of fasting. In an attempt to explain this correlation, we rule out anesthesia side-effects, handling, additional fasting, and desiccation. We use respirometric and metabolic techniques coupled with behavioral video scoring to assess energy expenditure in mounted and free flies. We isolate a specific behavior capable of exerting large amounts of energy in mounted flies and identify it as an attempt to escape from restraint. We present a model where physical restraint leads to elevated activity and subsequent faster nutrient storage depletion among mounted flies. This ultimately further accelerates starvation and thus increases reflexive feeding response. In addition, we show that the consequences of the physical restraint profoundly alter aerobic activity, energy depletion, taste, and feeding behavior, and suggest that careful consideration is given to the time-sensitive nature of these highly significant effects when conducting behavioral, physiological or imaging experiments that require immobilization.

## Introduction

Taste is an ancient chemosensory modality that allows distinguishing between helpful and harmful foods in our environment. Perception of taste is best characterized by the type of motivational response that it elicits^1–3^. These responses are traditionally classified as either appetitive or aversive^4–9^. Importantly, taste perception is modulated by collective interactions between sensory stimuli, internal satiety mechanisms, and previous experiences^10–18^. As such, when studying gustatory behavior, we must understand taste as a dynamic and multifaceted percept, rather than as a static and universal chemosensory response. An extensive arsenal of genetic techniques, numerous behavioral assays, and a relatively short life cycle makes *Drosophila melanogaster* a powerful insect model for studying gustatory behavior^6,19–24^.

When the tarsi or proboscis of a fruit fly comes in contact with a stimulus that is perceived as appetitive, proboscis extension may be elicited to initiate feeding^1,10,19,25,26^. This appetitive response can be used as a reliable index for motivational feeding behavior^1,10^. This technique has been used extensively to study numerous intrinsic and extrinsic factors that may drive motivational feeding behavior^27–31^. Intuitively, both the quality and concentration of the tastant affect its palatability, which suggests that the nature of the stimulus itself plays an important role in motivational feeding^2,32–36^. Further, aversive taste conditioning paradigms have demonstrated that associations established in prior experience play a significant role in mediating motivational feeding behavior^37,38^.

Motivational behavior is also mediated by internal signals representative of energy storage. The satiation state of a fly influences the amount of time that it spends foraging, which suggests that feeding behavior is in part driven by nutritional and metabolic states^39^. Furthermore, fasting increases appetitive behavior via changes in neuromodulatory states which are representative of internal nutrient deficits^12,20^, and even gut microbiota play a role in influencing behavioral feeding decisions^40^.

Many protocols that involve studying individual fruit fly behavior share a common set of preparatory techniques. It starts with removal from food, then anesthetization, and ultimately immobilization. The need for immobilization is common across a wide variety of assays that are concerned with real-time, single fly observation. Fly immobilization is necessary to perform live imaging studies^41^, electrophysiological recordings^42^, and behavioral studies^27^. Common methods of immobilization include adhesion using myristic acid^9^ nail polish^38^, lodging in a pipette tip^1,14^, or use of a fly collar^43^. Notably, each of these methods rely on physical restraint to immobilize the fly.

When using any paradigm for behavioral measurement, it behooves us to consider the effects of preparatory techniques such as immobilization on the behavior of the subject. This is particularly important when studying behavior, as these effects often have the capacity to confound the measurements of the behavioral phenotype of interest. Given the numerous factors that contribute to motivational feeding behavior, it is reasonable to consider that immobilization could unforeseeably impact feeding behavior in and of itself via unforeseen physiological and behavioral consequences. Here, we report a correlation between the amount of time that flies spend restrained and partially immobilized and the cumulative change in a subsequent motivational feeding behavior over time. We use behavioral, respirometric, and metabolic techniques to elucidate the link between immobilization and increased motivational feeding response in single flies and find a possible mechanism of excessive energy expenditure as the cause of the increase. We propose a model of a stress and anxiety elicited by the restraint, possibly combined with starvation, that is self-maintaining and self-propagating, and is exhibited as a continuous and energetically costly escape-motivated behavior.

## Results

**Figure 1:**
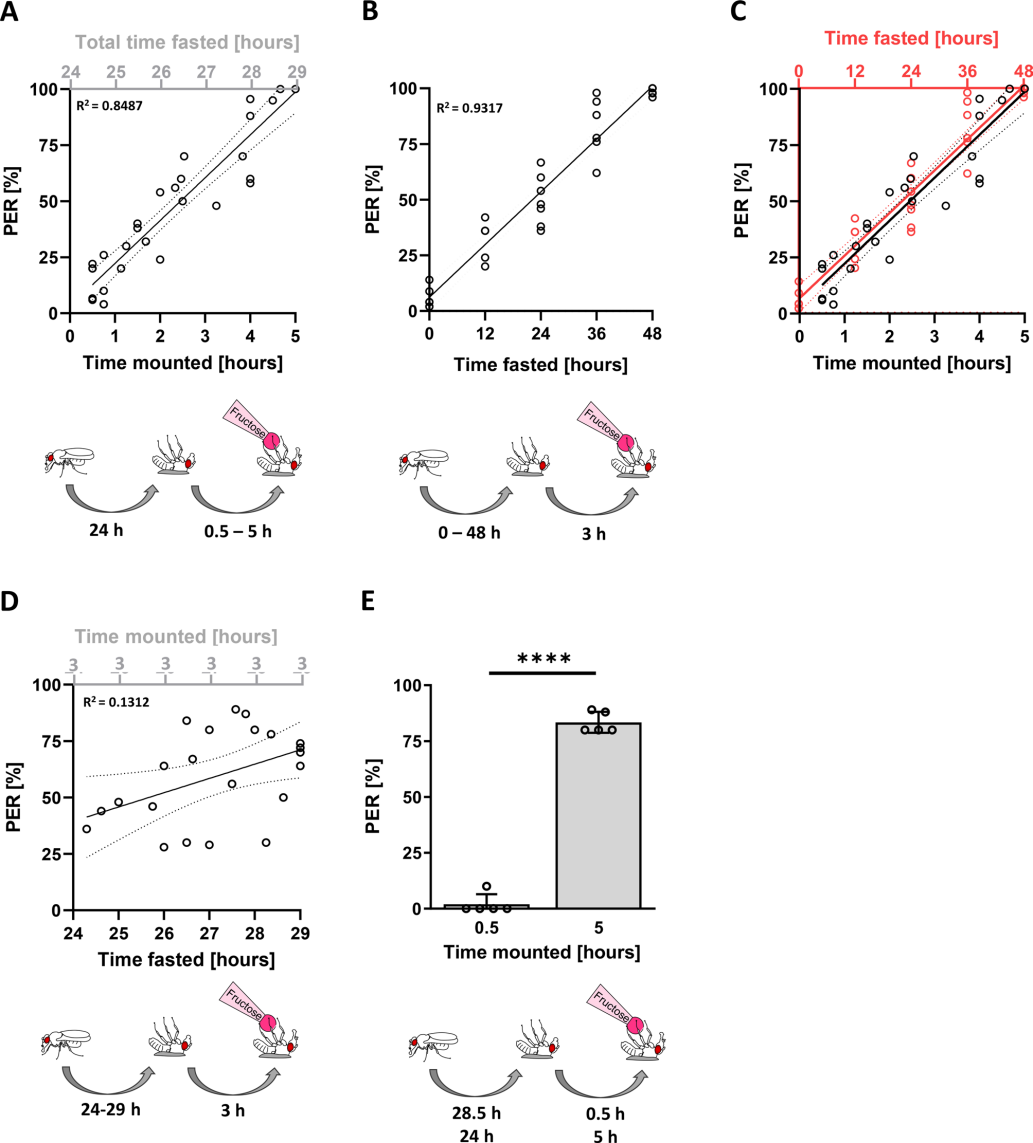
Flies mounted for longer time exhibit heightened motivational feeding response. (**A**) Average PER to 100 mM fructose of flies mounted between 0.5 and 5 h prior to testing (n = 29, N = 290) [simple linear regression]. (**B**) Average PER to 100 mM fructose among 0–48 h fasted flies after they were mounted for 3 h prior to testing (n = 30, N = 300). (**C**) Overlay of data from 1B with 1A shows an almost perfect overlap with nearly identical slope. (**D**) Average PER to 100 mM fructose among 24–29 h fasted flies that were all mounted for 3 h prior to testing (n = 23, N = 230). (**E**) Average PER to 100 mM fructose among flies fasted for 28.5 or 24 h, then mounted for either 0.5 or 5 h prior to measurement, respectively, with a total fasting time of 29 h in both groups (n = 5, N = 50 per condition ****P < 0.0001, t-test).

A Proboscis Extension Response (PER) assay offers an excellent measure of motivational feeding behavior, but to run this assay, flies must first be mounted on their backs to free their tarsi and proboscis. Typically, the amount of time allowed for flies to recover once mounted onto a microscopy slide prior to behavioral measurements (tastant presentation) is anywhere between 0.5 and 5 h. We found that there is an unexpected strong correlation between the length of time that flies are mounted prior to measurement, and the rate of PER (Fig. 1A, R^2^ = 0.8487, Y = 15.09X + 9.787). After five hours, when flies’ responses reached 100%, we reduced the concentration of fructose to 10 mM, to reduce the probability of PER, and we found that the increase continues with a similar slope as before, reaching 100% again after 5 additional hours of mounting (Fig. S1A). After fasting periods ranging from 0 to 48 h, we tested PER after 3 h of mounting (Fig. 1B, R^2^ = 0.9317, Y = 1.975X + 6.123). This is comparable to PER in flies mounted between 0 and 5 h after 24 h of fasting (Fig. 1C). This suggests that to change the rate of PER from approximately 0 to 100%, flies need either 5 h of mounting, or 48 h of being fasted. From this, it can be approximated that 1 h of mounting is comparable to approximately 10 h of starvation when tested for motivational feeding response.

### Assessing starvation, mounting, and anesthesia

Notably, flies that are mounted longer also experienced a longer period of food deprivation prior to PER measurement (from 24 to 29 h in Fig. 1A). To rule out the possibility that the correlation might be due to additional fasting, we staggered measurements such that all flies were measured after being mounted for 3 h, but were fasted between 24 and 29 h. The correlation between degree of fasting as an isolated variable and PER probability was far weaker, and the regression slope was much smaller (Fig. 1D, R^2^ = 0.1312, Y = 4.025X − 51.61). Furthermore, we found that flies mounted for 5 h show significantly higher rates of PER when compared to flies mounted for 0.5 h when both groups are measured after 29 h of fasting (Fig. 1E). This supports the notion that the correlation depicted in Fig. 1A is caused by factors other than fasting alone.

To mount flies, we must use an adhesive. It is possible that chemical or physical effects of adhesive administration may lead to a period of behavioral recovery where the rate of PER gradually increases. We use nail polish to mount flies. To mitigate possible effects of fumes, we mounted flies in a stream of fresh air. To completely avoid any aromatic chemicals, we also used a sealing wax and attached flies by melting a small portion of it. Neither the presence of ventilation, nor the use of a different adhesive substance altered the rate of PER measured at 3 h post mounting (Fig. S1B).

Since flies are anesthetized with carbon dioxide (CO_2_) prior to mounting, it is possible that CO_2_ exposure, anesthesia and the following recovery might affect behavioral responses, as was shown in other behaviors^44^, and might result in metabolic changes^45^. To rule out side effects of CO_2_, we mounted flies using cold anesthesia. We built a custom Peltier element cooling surface that allowed us to gently cool flies at a temperature of 10 °C. Flies recover from this anesthesia within several seconds after mounting as opposed to a CO_2_ recovery of several minutes. Yet, the cold anesthesia did not alter the increase of PER over time; and the correlation and regression slope remained unchanged (Fig. S1C, R^2^ = 0.8436, Y = 4.025X − 51.61). However, the possibility remained that both modes of anesthetization produced similar recovery effects. To rule this out, we re-anesthetized flies with CO_2_ after 0.5, 2, or 4 h of being mounted, and measured PER 1 h later. If anesthesia recovery was responsible for the correlation, these flies would exhibit a rate of PER concordant with flies that had been mounted for 1 h in Fig. 1B. However, in establishing the length of time mounted as an isolated variable, we still found a strong correlation with the rate of PER (Fig. S1D, R^2^ = 0.4977).

### Metabolic measurements

**Figure 2:**
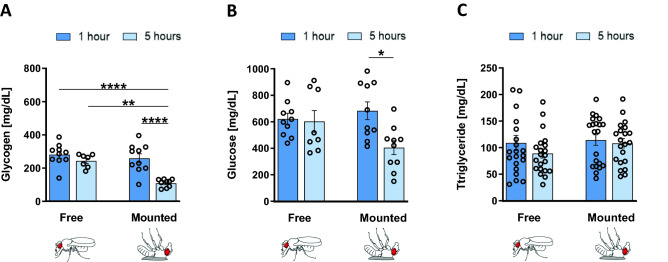
Flies mounted for longer experience depletion of glucose and glycogen storages. (**A**) Average glycogen (mg/dL) for free walking flies after 1 h (n = 10, N = 50) and 5 h (n = 8, N = 40) since anesthetization, and for mounted flies after 1 h (n = 10, N = 50) and 5 h (n = 10, N = 50) since mounting. (**B**) Average glucose (mg/dL) for free walking flies after 1 h (n = 10, N = 50) and 5 h (n = 8, N = 40) since anesthetization, and for mounted flies after 1 h (n = 10, N = 50) and 5 h (n = 10, N = 50) since mounting. (**C**) Average triglyceride (mg/dL) for free walking flies after 1 h (n = 21, N = 105) and 5 h (n = 21, N = 105) since anesthetization, and for mounted flies after 1 h (n = 21, N = 105) and 5 h (n = 21, N = 105) since mounting [*P < 0.05, **P < 0.01, ****P < 0.0001, ordinary one-way ANOVAs followed by Tukey’s multiple comparisons tests].

It has been demonstrated that increased hunger is reflective of internal energy storage depletion^39^. Since flies that were mounted for longer exhibited increased motivational feeding behavior, we reasoned that this difference in satiety state should also be reflected in the levels of energy storage. Indeed, flies that were mounted for 5 h demonstrated significant glycogen depletion compared to flies that were mounted for only 1 h (Fig. 2A, p < 0.01). Further, flies that were mounted for 5 h had significantly lower glycogen levels than flies that were free walking for 5 h after anesthesia (Fig. 2A, p < 0.05). These data show that glycogen is depleted significantly faster among mounted flies when compared to free walking flies, suggesting that there is an additional factor among mounted flies that leads to faster energy depletion.

We also compared glucose levels between flies that were mounted for 1 h with flies mounted for 5 h, the latter of which had significantly lower glucose levels (Fig. 2B, p < 0.05). On the other hand, free walking flies that were measured at 1 and 5 h after anesthesia administration showed no significant differences with respect to glucose levels (Fig. 2B). This suggests that mounted flies deplete their glucose at a faster rate than free walking flies, albeit the difference in glucose levels between free walking flies and mounted flies at 5 h was not significant. This is probably because hemolymph glucose is being continuously replenished from glycogen. As hemolymph glucose needs to be kept within a normal range of values, glycogen is likely converted to glucose when energy demand increases.

We saw no significant differences in triglyceride levels among flies mounted for 5 h, flies mounted for 1 h, or free flies (Fig. 2C, p < 0.05), indicating that the increase in the rate of PER among mounted flies is dependent on glycogen but not triglyceride storage depletion.

### Activity and respirometry

**Figure 3:**
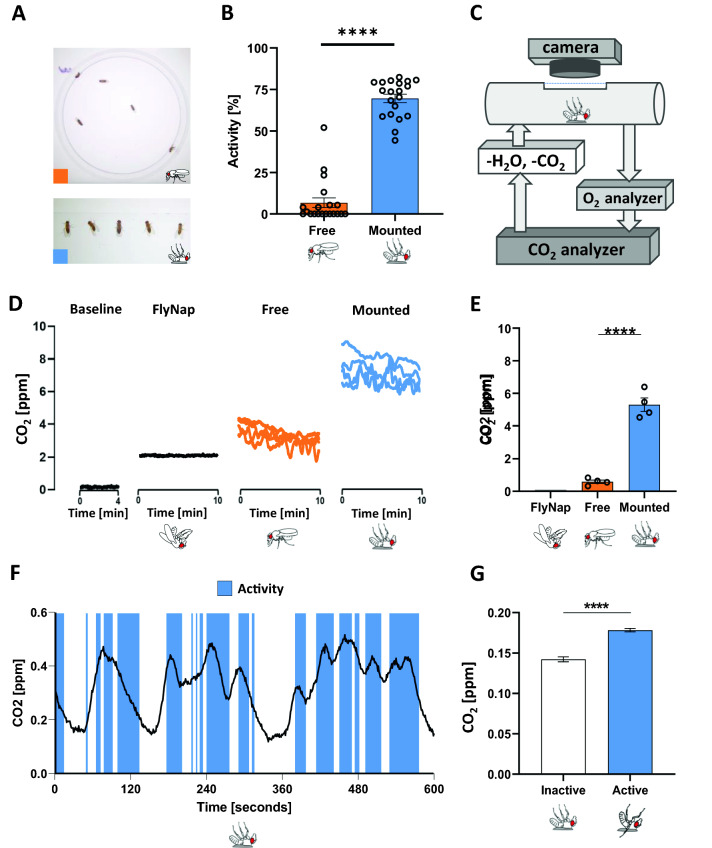
Mounted flies aerobically exert themselves more than free walking flies. (**A**) The activity of free walking and mounted flies were manually scored over 5 min. (**B**) Fraction of time spent active for free and mounted (n = 20, per condition) flies over 5 min, measured midday (**C**) Open-flow respirometry setup with live video recording. (**D**) Average CO_2_ production over a 10-min interval in free walking (n = 4, N = 40) and mounted flies (n = 4, N = 40), compared to machine baseline (n = 5), and FlyNap anesthetized flies (n = 4, N = 40). (**E**) Average CO_2_ production in free walking (n = 4, N = 40) and mounted flies (n = 4, N = 40) normalized to average CO_2_ production in FlyNap anesthetized flies (n = 4, N = 40) (**F**) Sample of data for continuous CO_2_ production in a single fly with overlayed bars representing activity bouts (n = 1). (**G**) Average CO_2_ production in single flies during periods of inactivity and during bouts of activity (N = 4). [****P < 0.0001 unpaired t tests].

Free walking flies tend to spend most of their time inactive and thus consume relatively little energy compared to, for example, flying flies^46–49^. We video-recorded free walking flies placed in a small chamber and flies mounted on a slide, and quantified their activity at midday (Fig. 3A, see Methods), and found that mounted flies spend significantly more time being active (Fig. 3B, p < 0.05). To assess the possible confounds of circadian rhythm on activity, we measured flies in the morning and observed the same (lack of) activity in free flies, but lower in mounted flies compared to midday, however, at both times, mounted flies spent significantly more time active compared to free flies (Fig. S2A, p < 0.0001). Starvation-induced hyperactivity has been previously shown in free-walking flies^50^. To assess whether the activity increase in mounted flies is also fasting dependent, we measured the activity of mounted fed flies and flies fasted for 1 or 2 days at 1 h after being mounted. We found no significant difference in activity as a function of starvation (Fig. S2B).

Next, we used an open-flow respirometer to measure CO_2_ production in groups of ten flies that were either free walking or mounted (Fig. 3C). Flies that were mounted produced a significantly higher amount of CO_2_ per second compared to free walking flies (Fig. 3D and Fig. S2, p < 0.05). Besides CO_2_, FlyNap, with an active ingredient of triethylamine, is another anesthetic commonly used to anesthetize *Drosophila*^51^. Unlike CO_2_ or cold anesthesia, administration of triethylamine seizes all major motor activity in flies, but maintains normal respiratory function^52^. Respirometric measurements of flies anaesthetized using triethylamine provide a baseline rate of CO_2_ production and metabolism of a fly without any major movement. We believe that subtracting this baseline measurement from both free walking and mounted flies is an even better representation of their difference in CO_2_ production (Fig. 3E, p < 0.0001). These findings suggest that the higher activity of mounted flies results in increased metabolic rate and thus in higher CO_2_ production. To better link the mounted fly activity and CO_2_ production, we scored the activity of an individual fly mounted in the respirometer while simultaneously monitoring its levels and CO_2_ production (Fig. 3C). We found that the bouts of activity in mounted flies tends to correspond with increases in CO_2_ production and that the overall production of CO_2_ during bouts of activity is significantly higher compared to bouts of inactivity (Fig. 3F,G, p < 0.0001). This supports the notion that higher activity demands more energy which is manifested in greater CO_2_ production.

### Escape-related behavior

**Figure 4:**
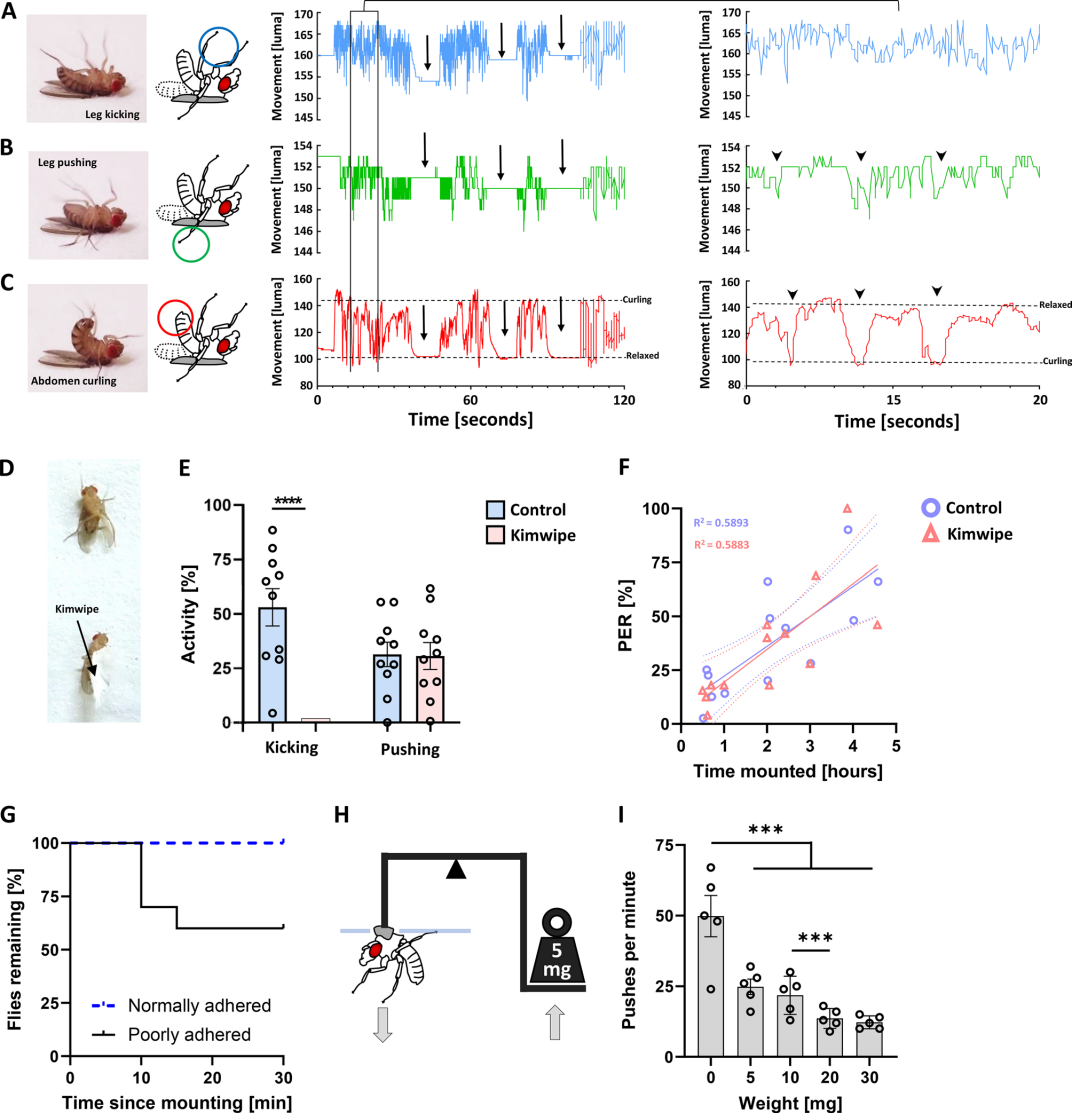
Leg pushing behavior is accountable for increased motivational feeding in restrained flies. (**A**) Motion detected in region signifying leg kicking behavior in mounted flies, graphically represented by average luminosity in the region over time (n = 1). (**B**) Motion detected in region signifying leg pushing behavior in mounted flies, graphically represented by average luminosity in the region over time (n = 1). (**C**) Motion detected in region signifying abdomen curling behavior in mounted flies, graphically represented by average luminosity in the region over time (n = 1). Arrows show times of inactivity where all behaviors cease. Arrow heads show correlation of an individual leg pushing and abdomen curling. (**D**) Flies are given a small piece of Kimwipe paper to be held by their tarsi. (**E**) Average kicking and pushing activity levels for flies with and without pieces of Kimwipe on their tarsi (n = 10, N = 100 per group). (**F**) Average proboscis extension reflex (PER) in flies with and without pieces of Kimwipe on their tarsi after 0.5–5 h of being mounted (n = 13, N = 130 per condition). (**G**) Flies remaining over 30 min after being normally adhered, and poorly adhered (n = 10, N = 100 per condition, p = 0.0289, survival curve comparison test). (**H**) Balanced ‘seesaw’ apparatus which allows individual flies to raise weights during leg pushing behavior (**I**). Average number of pushes per minute across a range of weights (N = 6). [***p < 0.001*,* ****p < 0.0001, unpaired t tests].

The activity of mounted flies, as previously compared to the activity of free walking flies, was scored by observing the presence or absence of leg movements. Closer examination revealed that this activity consisted of three unique behaviors which could be separately assessed: leg kicking, leg pushing, and abdomen curling. When observing a mounted fly carefully, these three behaviors are continuously, and often simultaneously present. We used software to measure changes of pixel light intensity in specific regions of interest on high resolution videos which were positioned to selectively detect each of these three behaviors (Fig. 4A–C). This qualitative tracking demonstrates that leg kicking, leg pushing, and abdomen curling can each be assessed independently; but that there is noticeable temporal overlap at low and high time resolution (Fig. 4A–C). It is possible that it is the collective effort of these three behaviors that is responsible for the energy expenditure previously shown. To assess whether one or more of these behaviors is responsible for energy storage depletion, we asked whether preventing flies from kicking their legs would reduce the energy expenditure over time. A small piece of tissue paper (Kimwipe) was placed on fly tarsi (Fig. 4D). This prevented leg kicking behavior while maintaining leg pushing and abdomen curling activity (Fig. 4E). Eliminating leg kicking did not alter the correlation between the length of time mounted and the rate of PER (Fig. 4F, R^2^ = 0.5893, Y = 13.89X + 8.359 for control, R^2^ = 0.5883, Y = 15.25X + 4.144 with Kimwipe). This leaves the leg pushing and abdomen curling as the behaviors accountable for energy expenditure in mounted flies. We speculate that these particular behaviors were instances of escape attempts among restrained flies. In fact, flies mounted to microscopy slides by an inexperienced experimenter often push themselves free with what appears to be significant force. We attempted to reproduce these conditions of poor attachment by using partially dried nail polish. The flies that were attached poorly indeed demonstrated a significantly higher likelihood of successfully peeling themselves from the slide and escaping (Fig. 4G, p < 0.05). By escaping and righting themselves, these flies almost instantly stopped moving and remained relatively stationary, much like the free walking flies we recorded in the previous experiments (video not shown). This suggests that the restrained flies, whether successful or not, likely put in a significant effort to escape, which is a parsimonious explanation for frequent pushing on the microscopy slide while restrained. It has been shown that insects are capable of generating enormous amount of force relative to their body weight^53,54^ and even *Drosophila* is capable using 15 times more energy while flying than when walking^22^. To estimate the amount of force that a fly is capable of using while pushing against the slide they are attached to, we built a balance (‘seesaw’) apparatus which allows us to measure how much weight a fly can lift in a position that is similar to how they are mounted. Flies were mounted to a bent pin and placed between two cover slips such that their legs were able to push against them upwards (while the fly was mounted facing down). On the other side of the pin, we hung metal weights of increasing heaviness (Fig. 4H). We scored the number of pushes that moved the fly noticeably away from the slides for one minute (downward arrow in Fig. 4H). Without any added weight, the pin was balanced so that when pushing, flies were moving negligibly more than their own body weight. With no additional weight, they pushed with a very high frequency, close to one push per second (Fig. 4I). The frequency decreased significantly with an additional 5 mg, and further with 20 mg (Fig. 4I). Our apparatus failed to hold with higher weights, however, considering that the decrease in number of pushes between 20 and 30 mg was minimal, it is likely that flies are capable of pushing even more weight. Even so, 30 mg weight is nearly forty times the fly’s body weight (female, 0.776 mg N = 80). It is thus likely that pushing is the major source of nutrient storage depletion during prolonged periods of restraint.

## Discussion

We described an effect that acute physical restraint in mounted flies has on subsequent measures of motivational feeding behavior and energy storage levels. This study shows that restraint, an unavoidable part of fly mounting, leads to behavioral changes that can interfere with subsequent behavioral and physiological measurements^2,38,55^. We speculate that any behavioral measure that is dependent on level of fasting will be sensitive to this effect. It has been shown that there is an interplay between olfaction and feeding response^56^, that sleep and sleep deprivation is dependent on satiation^50,57^, and that the formation and persistence of memory depends on the level of perceived sweetness^58–60^. As such, we expect that any measurements relevant to these areas of study are susceptible to confounding starvation effects caused by restraint. Even behaviors that do not have a direct link to taste and feeding can be influenced by the excessive energy loss. Flight in flies is very energetically demanding^49,61^ and mounting flies to flight simulators, often for over 12 h before measurement, could lead to greater energy depletion, a reduction or alteration in the fly’s ability and willingness to fly, and a decrease in ability to perform learning or more complex cognitive tasks^62,63^.

There are a multitude of mounting techniques used to study behavior in flies, none of which we believe are entirely immune to the effects we discuss. One common technique used to study fly walking and navigation involves mounting a fly to a ball suspended on air^64–67^. For both flight and walking experiments, flies are attached either to a solid platform, similar to the way we mounted flies in this study, or they are mounted to a thin metal hook between their head and thorax^62,68,69^. Even flies that are mounted to a hook attempt to pull on it with their legs and will occasionally remove themselves in a manner that is similar as depicted in Fig. 4G. We can assume that the escape-related behavior likely uses similar force as is described in this study; and are consequently energetically taxing. Many of these mounting techniques where flies are glued by their thorax, head, or both are also widely used for calcium imaging^4,68,70,71^. If the mounting period is not long, the effects of energy depletion due to restraint-induced struggle may not be significant, however for assays where recording for longer than a few minutes is necessary, confounding effects are possible. In some assays, mounting time can be as long as 12–24 h^2,72,73^, and because we show the effect is constant for a minimum of 10 h, careful attention should be paid to mitigate this nonspecific variable, especially in these assays.

Some mounting techniques, namely placing flies inside a pipette tip, do not require the use of an adhesive^1,9^. Nevertheless, we assume that, if measured and closely observed, the flies would display signs of struggle, and would likely exert extra energy despite their inability to move their limbs and abdomen along visible trajectories. In such assays where behavioral activity cannot be measured, the depletion of energy storage could be used to assess the degree of struggle in different mounting preparations. In preparations where enclosure in a metabolic chamber is possible, CO_2_ could be measured in real time to estimate the energy expenditure of mounted flies^48^.

It might seem that using assays where flies are not mounted could eliminate effects associated with the fly’s attempt to free itself. However even in some assays where the fly is free to move, but is restricted by walls or water barriers, there is still an element of entrapment that might motivate attempts to escape^47,50,74,75^. There is no doubt that flies continuously try to escape from restraint or confinement; and anyone who has ever worked with flies has experienced that they often succeed in doing so. In some situations, where escape is impossible, flies exhibit learned helplessness and no longer attempt to escape^76,77^. We have not observed any decrease in escape-related behavior over the course of 10 h, suggesting that this form of restraint does not result in learned helplessness. Flies that are ‘trapped’ by visible or invisible walls continue to walk for hours, whereas those in relatively free environments tend to spend most of their time standing and grooming^47,78,79^. It would be interesting to measure the extent to which spatially confined, but free walking flies exert energy in their attempts to escape.

The reported effect of CO_2_ anesthesia on locomotor behavior depends on the length of time flies spend on the CO_2_ pad and the volumetric flow rate of CO_2_^44^. It is possible that we did not see any effect of anesthesia because our mounting technique is fast (approximately 1 min per slide), and the flow rate of CO_2_ we use is low. Additionally, we use an in-line water bath to humidify the CO_2_ and reduce desiccation, which, along with CO_2_-specific mechanisms and an anoxic effect, causes behavioral deficits after anesthesia^44^. Furthermore, an alternative anesthetic did not alter the correlations (Fig. S1C), supporting the notion that anesthesia, despite its effect on metabolic processes, does not play role in the described effect^45^.

Physical restraint produces an increase of energetically costly physical activity in the form of leg pushing and abdomen curling. This is perhaps a fly’s attempt to free itself from restraint, as well as to right itself and regain contact of its tarsi with the ground. Female flies used in our experiments weighed an average of 0.776 mg each, but were capable of moving a 30 mg weight every 6 s. Although this is not as impressive as the strongest reported insect^53^, female flies were still able to lift almost 40 times their body weight repeatedly. We were not able to quantify the energy needed for these lifts, as the weights were moved a very short distance, and leg flexion would further complicate the calculations. However, considering that a standing fly maximally holds its own weight, these data suggest that leg pushing during restraint is highly energetically taxing, even if the behavior is exhibited only about 30% of the time (Fig. 4E).

It is possible that the sustained and elevated activity is caused by stress from being restrained. Stress might parsimoniously explain the motivation underlying the persistent, though often unsuccessful attempts to escape from their restraint. A correlative link between acute restraint and elevated locomotor activity was previously described in mice^80,81^. It was suggested that this relationship is mediated by pathways associated with anxiety behavior^82,83^. Serotonin (5-HT), dopamine and octopamine play an essential role in stress and anxiety in mammals^84–86^ and were all reported to be involved in similar behaviors in flies^87–90^. Therefore, it is possible that neurotransmitters involved in anxiety might mediate the restraint-related behaviors described in this study. Future correlative investigation into the link between restraint, feeding, and energy expenditure in flies may present a novel way to model and study anxiety in invertebrate species.

**Figure 5:**
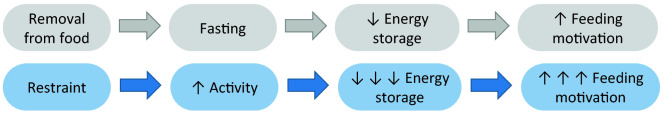
Fasting and restraint additively leads to excessive storage depletion and consequently to elevated feeding motivation. Removal of flies from food leads to fasting that, over time, uses energy storage which can be assessed by measuring a feeding motivation response. Acute physical restraint leads to extended bouts of activity during which we observe elevated levels of energetically demanding behaviors (leg pushing and abdomen curling). This activity depletes internal energy storage far more rapidly than fasting alone, which in turn elevates the internal hunger state and motivational feeding response at a comparatively faster rate. Consequently, the combination of fasting and restraint produces a rise in motivational feeding response over time that is ten times more rapid than from fasting alone.

Based on our data, we propose a model explaining the correlation between the length of time mounted and an increase of motivational feeding response (Fig. 5). When mounted flies attempt to free themselves, they use force that is more than an order of magnitude greater than what is necessary for standing or walking. During extended bouts of activity, the use of this excessive force results in rapid depletion of energy storages such as glycogen. This loss of energy elevates their internal hunger state which manifests as an increase in motivational feeding response.

We show that physical restraint profoundly alters aerobic activity, energy depletion, and feeding behavior. The combination of fasting and restraint produces an increase in motivational feeding response that occurs ten times more rapidly than from fasting alone. This indicates that even a short mounting time can profoundly affect many behavioral, physiological, or imaging experiments requiring immobilization.

## Materials and methods

### Experimental animals

*Drosophila melanogaster* Canton-S flies were grown and maintained on standard food media based on cornmeal/molasses (Jazz mix, Fisher Scientific) and kept in an LD incubator (Powers Scientific; Dros52) at 25 °C and 60% humidity on a 12:12 light–dark cycle. For all experiments, 3–5 days old, mated females were used. All experimental and control groups were measured in parallel at the same Zeitgeber time during midday.

### Motivational feeding assay

Flies were fasted for 24 h prior to preparation for proboscis extension reflex (PER) assay. Flies anesthetized using CO_2_ were sorted out and size matched females were adhered by their dorsal side of their thorax to a microscopy slide by using nail polish (Cat#72180, Electron Microscopy Science) as described previously^2,38^. After varying lengths of recovery time, PER was measured as follows: flies were water satiated and were used for experiments only after not responding to water three consecutive times. Flies which continued responding to water after 5-min were excluded from the experiment. Then, 10 mM or 100 mM fructose (CAS#57-48-7, Sigma Aldrich) was applied briefly (1 s) to the tarsi and the proboscis extension was recorded (only binomial yes/no response was recorded). Each tastant was presented five consecutive times with water used for tarsi washing and satiation (as needed) between each trial. 1 M sucrose (CAS#57-50-1, Sigma Aldrich) was applied at the end of each session to check for the responsiveness of each fly. Flies that did not respond to sucrose at the end were excluded. An index of PER response was calculated as a percentage of proboscis extensions out of the total number of tastant presentations per fly and per group.

### Tests for effects of anesthesia and adhesive

Experiments were performed to test effects of different anesthetics used cold-plate exposure at 10 °C for 30 s to anesthetize flies prior to mounting. To test the effects of CO_2_ anesthesia on PER, 3–5 days old, mated females were fasted for 24 h, then anesthetized using CO_2_ exposure prior to mounting for 0.5, 2.5, or 4 h. Flies were then anesthetized again using CO_2,_ carefully scraped from the microscopy slide, re-mounted, then tested for PER in response to a 100 mM fructose solution after one additional hour of being mounted.

Otherwise, PER was performed as outlined above. Experiments performed to test the effects of different adhesives used slow airflow using aquarium pump across each microscopy slide as flies were mounted using nail polish, then tested for PER with 100 mM fructose solution after 3 h of being mounted. Flies were alternatively mounted by applying strips of sealing wax (XICHEN, ASIN—B00Y24YJRU), allowing it to dry, then melting small sections to mount individual flies.

### Respirometry

Flies were fasted for 24 h prior to preparation for respirometry protocols. For mounted flies, flies were anesthetized using CO_2_ and adhered to a microscopy slide using nail polish as performed in the PER preparatory procedure. Flies were allowed to recover for varying lengths of time and then placed in an open-flow respirometer. Free flies were anesthetized using CO_2_ and placed in a humidified vial for varying lengths of time. These flies were transferred to a custom-made observation chamber using mouth aspirator and placed inside an open-flow respirometer. After placed inside of the CO_2_ chamber, flies were allowed to settle for 5 min and only the following 10 min were used for measurements. CO_2_ production and video taken through window in the CO_2_-recording chamber were recorded simultaneously.

### Behavioral activity scoring

Individual fly behavior was manually scored per second as either active or inactive. Mounted flies that were moving their legs (kicking) or were pushing against the slide (correlated with extreme abdomen curling), were scored as active. Free walking flies that were walking or running were scored active and those still or grooming were scored as inactive. Tracker software (https://physlets.org/tracker) was used to distinguish three distinct behaviors in mounted flies (leg kicking, leg pushing, abdomen curling) by monitoring average luma in each selected region of interest (ROI) over time as outlined in Fig. 4A–C.

### Leg kicking restriction experiment

Flies were fasted for 24 h and were anesthetized and mounted as previously described. These flies were split into two groups. In one group, each fly was given a torn piece of Kimwipe paper (approximately 2 × 2 mm) to be held by their tarsi; while the control group remained free to kick their legs. Both groups were placed in a humidified chamber and monitored by an observer continuously. Any fly that dropped their Kimwipe paper had it immediately placed back on their tarsi. Any disturbances or removals from humidified chamber were mirrored in the control group. Manual behavioral activity scoring was performed as described above. PER to 100 mM fructose solution was measured in both groups of flies after varying lengths of time mounted, as described previously.

### Escape experiment

Flies were fasted for 24 h and were then split into two groups. One group was adhered with nail polish normally as previously described. The second group was purposefully adhered poorly by allowing each spot of nail polish on the microscopy slide to dry for approximately 5 s prior to mounting each fly. The number of flies remaining on the microscopy slide for each group was monitored over 30 min.

### Metabolic assay fly preparation

Flies were collected and placed on fresh food for 24 h, then starved for 24 h in food-vials on wet Kimwipe paper. Flies anaesthetizing on CO_2_ pad and groups of five flies were adhered onto each microscopy slide. The combined weight of each microscopy slide plus five flies was recorded after gluing. Flies were left to recover in a box with wet paper towel for either 1 h or 5 h. After mounted time elapsed, flies were given water until satiation and then were weighed again. This mostly led to equal weight measured directly after gluing suggesting that weight lost during gluing was mostly due to desiccation. Flies were then anaesthetized and scraped off the microscopy slide using a razor blade and frozen in 1.5 mL Eppendorf tubes.

Free-walking control flies were anaesthetizing on CO_2_ pad and groups of five flies were placed in vials on wet Kimwipe paper for either 1 h or 5 h. Flies were then anaesthetized and frozen in 1.5 mL Eppendorf tubes.

### Glucose, glycogen, and triglyceride analysis

Eppendorf tubes with five flies were kept on ice until the flies were homogenized in 100 μL of phosphate-buffered saline (PBS). The samples were centrifuged at 13,000 rpm for 10 min at 4 °C to remove body parts and flakes of nail polish. The supernatant and top fatty layer of each sample was transferred to a new 1.5 mL Eppendorf tube. Fat was resuspended in the supernatant by vortexing, creating a crude suspension of cell lysate.

For glucose analysis, 2 μL of crude lysate for each was added to 98 μL of Infinity Glucose reagent (Cat#TR15421, Thermo Scientific) in a 96-well plate. The plate was incubated at 37 °C for 5 min, then read at 340 nm. Readings were compared to standard solutions of 0, 25, 50, 100, 250, 500, 1000 mg/dL glucose.

Glycogen assays were run with the same samples simultaneously with the glucose assay, on the same 96-well plate. 1 μL of amyloglucosidase (Sigma A1602-25MG) was added per mL of Infinity Glucose reagent in wells intended for glycogen analysis. 2 μL of each lysate sample was added to 198 μL of amyloglucosidase and Infinity glucose (or 2 μL of lysate to 98 μL of plain Infinity glucose reagent) mixture. The plate was incubated for 5 h at 37 °C and read 340 nm. Readings were compared to standard solutions of 0, 25, 50, 100, 250, 500, 1000 mg/dL glucose.

For triglyceride analysis, Eppendorf tubes with five flies were kept on ice until the flies homogenized in 200 μL PBS + 0.1% Tween. Homogenized samples were heated at 65 °C for 5 min to inactivate lipases. 2 μL of crude lysate and add to 198 μL of Infinity TAG reagent (Cat#TR22421, Thermo Scientific) for each sample in a 96-well plate. Samples were incubated at 37 °C for 5 min then read in a plate reader at ~ 540 nm. Readings were compared to standard solutions of 0, 10, 50, 100, 150, 200, 400, 600 mg/dL TAGs. Cayman Chemical (Item #10010509) was used for this assay.

### Statistical analysis

Values for all experiments are displayed as mean ± SEM with individual values showed where possible. Unpaired t-test was used to test for significance from zero. Paired t-test was used to test for significance between two groups. Survival curve comparison was performed using the Mantel-Cox (log-rank) test. One-way ANOVA with tests of multiple comparisons were performed to compare groups in all assays. Statistical analyses and data presentation were performed using Prism software (GraphPad Software 8.0; San Diego, CA, USA).

### Reproducibility statement

For all experiments, ‘n’ denotes the number of independent measures, while ‘N’ represents the total number of experimental animals. All major experiments were repeated on two or more non-consecutive days to assure reproducibility of results (Figs. 1A–D, 2A–C, 3D–G, 4F, Fig. S2B,C).

## Supplementary Information

Supplementary Information.
