# Supplementary material for: Excessive energy expenditure due to acute physical restraint disrupts Drosophila motivational feeding response
Source: Sci Rep. 2021 Dec 17;11:24208. doi: 10.1038/s41598-021-03575-3 (PMC8683507; doi:10.1038/s41598-021-03575-3)
Supplement: Supplementary file 1 — Supplementary Information. [file 41598_2021_3575_MOESM1_ESM.pdf]

Excessive energy expenditure due to acute physical restraint disrupts *Drosophila* motivational feeding response

Jacob Gordon[1], Pavel Masek[1]

[1] Department of Biological Sciences, Binghamton University, 4400 Vestal Parkway East, Binghamton, NY 13902

P.M. +1 607 777 6374, flytaste@gmail.com

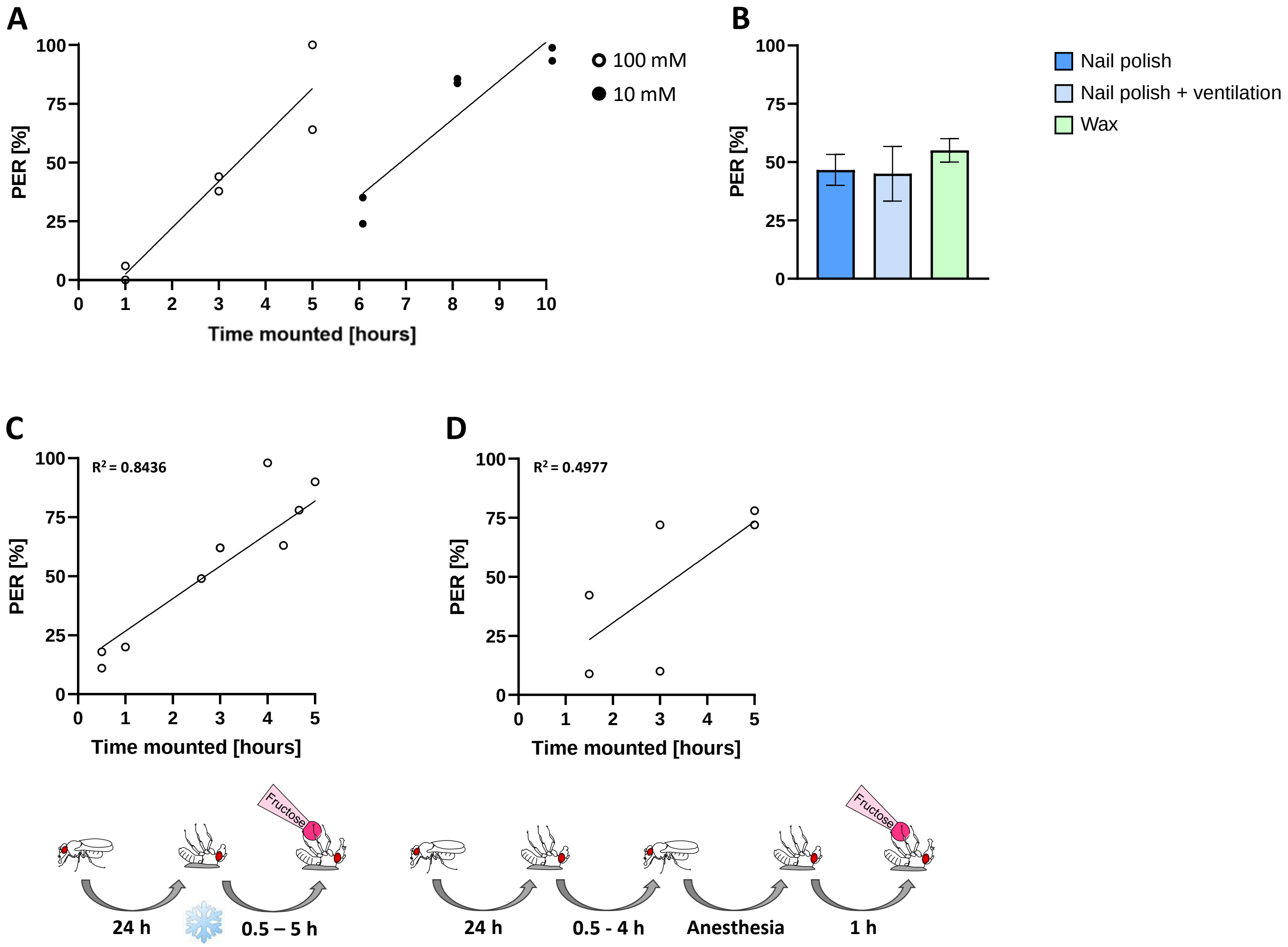

Supplementary Figure 1.

Anesthesia recovery nor adhesive effects can account for heightened motivational feeding response.

(**A**) Average PER in response to 100 mM fructose among 25-29 hour fasted flies mounted between 1 and 5 hours (n = 6, N = 60), combined with average PER in response to 10 mM fructose for 30-34 hour fasted flies mounted between 6 and 10 hours (n = 6, N = 60). **(B)** Average PER in response to 100 mM fructose among 27 hour fasted flies that were mounted for 3 hours using nail polish, nail polish with moving air ventilation, or wax (n = 2, N = 20 per group). (**C**) Average PER in response to 100 mM fructose among 24-29 hour fasted flies that were anesthetized using cold exposure, then mounted between 0.5 and 5 hours prior to testing (n = 13, N = 130) [$R^2 = 0.8436$, simple linear regression]. (**D**) Average PER to 100 mM fructose among 24-29 hour fasted flies that were mounted between 0.5 and 4 hours prior to being removed, re-anesthetized, and remounted for an additional 1 hour prior to measurement (n = 6, N = 60) [$R^2 = 0.4977$, simple linear regression].

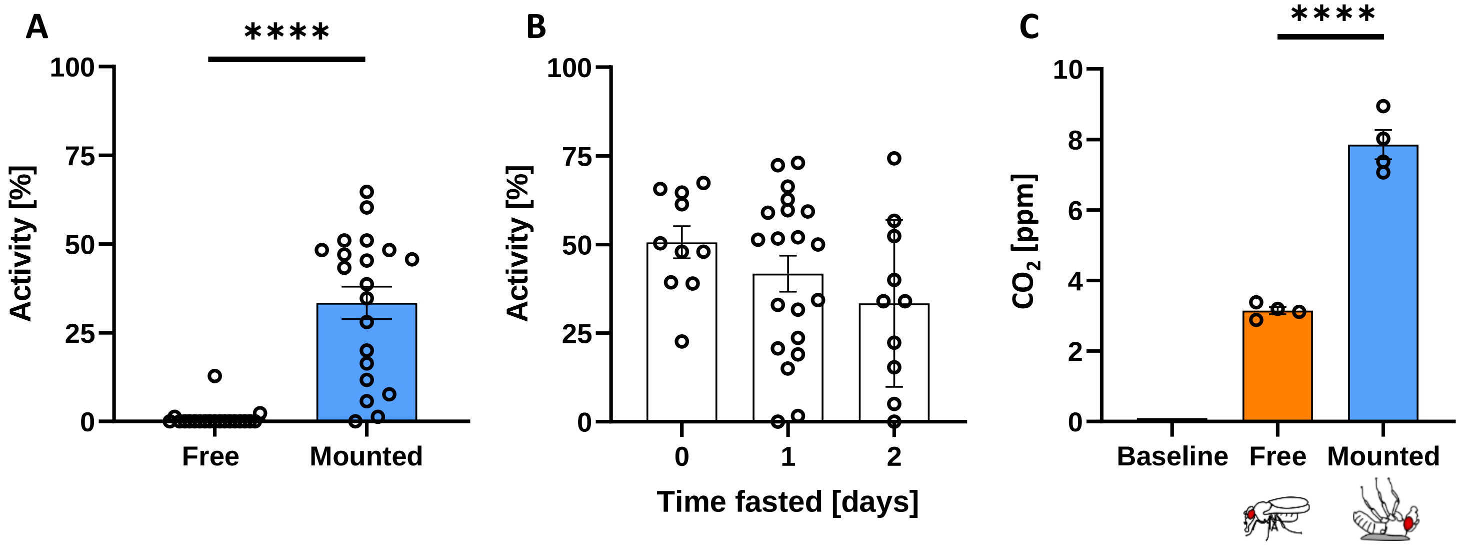

Supplementary Figure 2.
Mounted flies aerobically exert themselves more than free walking flies.
(**A**) Fraction of time spent active for free and mounted flies over 5 minutes, measured in the morning (n = 20, per condition) **(B**) Average activity of mounted flies that were fed (n = 10, N = 100), fasted for 1 day (n = 20, N = 200), or fasted for 2 days (n = 10, N =100) at 1 hour after being mounted. (**C**) Average CO_2 production in free walking (n = 4, N = 40) and mounted flies (n = 4, N = 40) normalized to machine baseline (n = 4, N = 40) [**** $P < 0.0001$, unpaired t test].
